# Zona incerta mediates early life isoflurane-induced fear memory deficits

**DOI:** 10.1038/s41598-024-66106-w

**Published:** 2024-07-02

**Authors:** Jing Sun, Xiaofei Deng, Lin Zhu, Jianbang Lin, Gaowei Chen, Yong Tang, Shanshan Lu, Zhonghua Lu, Zhiqiang Meng, Yuantao Li, Yingjie Zhu

**Affiliations:** 1 Department of Anesthesiology, Shenzhen Maternity and Child Healthcare Hospital, The First School of Clinical Medicine, Southern Medical University, Shenzhen, 518028 China; 2 The Brain Cognition and Brain Disease Institute, Shenzhen-Hong Kong Institute of Brain Science, Shenzhen Institute of Advanced Technology, Chinese Academy of Sciences, Shenzhen, 518055 China; 3 Department of Neonatology, Shenzhen Maternity and Child Healthcare Hospital, The First School of Clinical Medicine, Southern Medical University, Shenzhen, 518028 China; 4 University of Chinese Academy of Sciences, Beijing, 100049 China; 5 Biomedical Research Institute, Hubei University of Medicine, Shiyan, 442000 China

**Keywords:** Anesthesia, Cognitive development, Neonates, Isoflurane, Dexmedetomidine, Zona incerta, Fear conditioning test, Neuroscience, Physiology, Neurology

## Abstract

The potential long-term effects of anesthesia on cognitive development, especially in neonates and infants, have raised concerns. However, our understanding of its underlying mechanisms and effective treatments is still limited. In this study, we found that early exposure to isoflurane (ISO) impaired fear memory retrieval, which was reversed by dexmedetomidine (DEX) pre-treatment. Measurement of c-fos expression revealed that ISO exposure significantly increased neuronal activation in the zona incerta (ZI). Fiber photometry recording showed that ZI neurons from ISO mice displayed enhanced calcium activity during retrieval of fear memory compared to the control group, while DEX treatment reduced this enhanced calcium activity. Chemogenetic inhibition of ZI neurons effectively rescued the impairments caused by ISO exposure. These findings suggest that the ZI may play a pivotal role in mediating the cognitive effects of anesthetics, offering a potential therapeutic target for preventing anesthesia-related cognitive impairments.

## Introduction

The safety and potential long-term effects of anesthesia on cognitive development, especially in neonates and infants, have raised considerable concerns. While some clinical studies suggest potential risks associated with early anesthesia exposure and its impact on cognitive development in children^1–6^, others yield inconclusive results^7,8^. Additionally, numerous animal studies, including non-human primates, consistently suggest that specific anesthetic agents have the potential to trigger neonatal brain cell death, ultimately resulting in long-term neurocognitive dysfunction^9–12^.

Volatile inhalation anesthetics are commonly used in children and infants, and have been at the center of this debate. Evidence from neonatal rodents and monkeys has demonstrated that early exposure to isoflurane can induce neuroapoptosis, which is positively correlated with cognitive dysfunction in adulthood^13–15^. Importantly, animal studies have shown that the brain's susceptibility to anesthesia-induced damage is age-dependent, reaching its peak for most brain regions at a very early postnatal age (around 7 days old in rodents), while in certain brain areas, this peak may be delayed to the weanling stage (around 21 days in rodents)^16–18^. However, this phenomenon has not yet been confirmed in humans. Furthermore, early exposure to anesthesia appears not to significantly impact adult neuronal density or impair spontaneous locomotion, spatial learning, or memory functions^19,20^. These findings suggest that there might be a critical window for interventions targeting anesthesia-induced cognitive impairments.

Efforts have been made to mitigate the adverse effects of anesthetics. Clinical finding indicate that dexmedetomidine can reduce isoflurane requirements and its associated cognitive impairment in a dose-dependent manner^21^. In neonatal rats, dexmedetomidine can attenuate isoflurane-induced neuroapoptosis and long-term memory impairment^22^ when combined with isoflurane, dexmedetomidine appears to modify neuronal activity, suggesting a potential interaction between the two agents in mediating anesthesia and cognitive effects^23^. The precise mechanisms of this interaction, however, remain to be elucidated.

In the present study, we delve deeper into the effects of dexmedetomidine on isoflurane-induced cognitive anomalies, focusing on the underlying neural mechanisms in mice. Our findings indicate that neonatal isoflurane exposure hampers memory in adult, as evidenced by a fear conditioning test. Remarkably, pre-treatment with dexmedetomidine seems to counteract this impairment. We further identify the zona incerta (ZI) as a pivotal brain region influencing isoflurane-induced cognitive impairment and the protective role of dexmedetomidine.

## Results

### Isoflurane-induced fear memory deficit is reversed by dexmedetomidine

**Figure 1:**
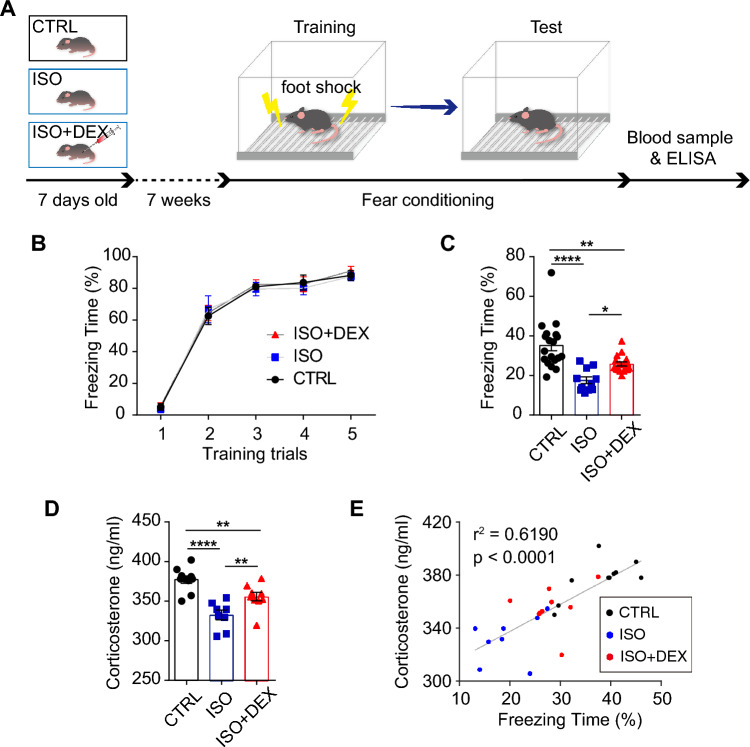
ISO-induced fear memory deficit is reversed by DEX. (**A**) Graphical representation of the sequence of behavioral paradigms and schematic illustrating neonatal ISO exposure and the adult fear conditioning paradigm. (**B**) Percentage of freezing time during fear conditioning training for control, ISO, and ISO + DEX groups. Control group (n = 19): 5.1% ± 8.9%, 62.6% ± 23.2, 81.0% ± 6.8%, 83.7% ± 20.3%, 88.2% ± 13.2%; ISO group (n = 11): 4.0% ± 7.9%, 67.0% ± 28%, 79.6% ± 13.8%, 80.3% ± 14.1%, 87.6% ± 9.0%; ISO + DEX group (n = 17): 6.0% ± 8.1%, 64.7% ± 20.0%, 82.5% ± 12.3%, 82.8% ± 19.1%, 91.1% ± 12.1%. (**C**) Percentage of freezing time during retrieval of contextual fear memory for the control, ISO, and ISO + DEX groups. One-way ANOVA, F(2, 44) = 15.85, *P* < 0.0001, followed by post hoc Fisher’s LSD test, *P < 0.05, **P < 0.01, ****P < 0.0001; (**D**) Corticosterone levels for the control (n = 10), ISO (n = 8), and ISO + DEX (n = 9) groups. One-way ANOVA, F(2, 24) = 17.22, *P* < 0.0001, followed by post hoc Fisher’s LSD test, **P < 0.01, ****P < 0.0001. (E) Correlation analysis of corticosterone levels and freezing time. Pearson correlation, r^2^ = 0.6191, ****P < 0.0001. All data are expressed as mean ± SEM.

To investigate the effects of early life anesthesia on later memory formation and retrieval, we exposed pups on postnatal day 7 (P7) to isoflurane (ISO) for 6 h and performed standard fear conditioning on postnatal day 56 (P56). To examine the effect of dexmedetomidine (DEX), another group of mice received an intraperitoneal injection of DEX (75 µg/kg) 20 min before the ISO anesthesia (Fig. 1A). During fear conditioning, control mice quickly acquired freezing behavior when the tone was on, suggesting they learned the association between the tone and foot shock. Mice exposed to ISO (ISO group) and those additionally treated with DEX (ISO + DEX group) showed similar levels of freezing behavior compared to the control mice (Fig. 1B). These results indicated that ISO and DEX did not affect the acquisition of fear memory. Twenty-four hours later, when the mice were re-exposed to the same context during the training (retrieval of fear memory), control mice exhibited a significant portion of freezing behavior. The ISO group showed a significant reduction in freezing time compared to the control group, suggesting the impairment of fear memory by early life ISO anesthesia (Fig. 1C). Treatment with DEX increased the percentage of freezing time compared to the ISO group, suggesting a protective role of DEX on ISO-induced fear memory deficits. The ISO group showed reduced serum corticosterone compared to the control group, while the ISO + DEX group exhibited increased corticosterone levels compared to the ISO group (Fig. 1D). Moreover, the corticosterone level was correlated with freezing time (Fig. 1E).

### *ISO* induced neuronal activation in the *hippocampus* and the zona incerta

**Figure 2:**
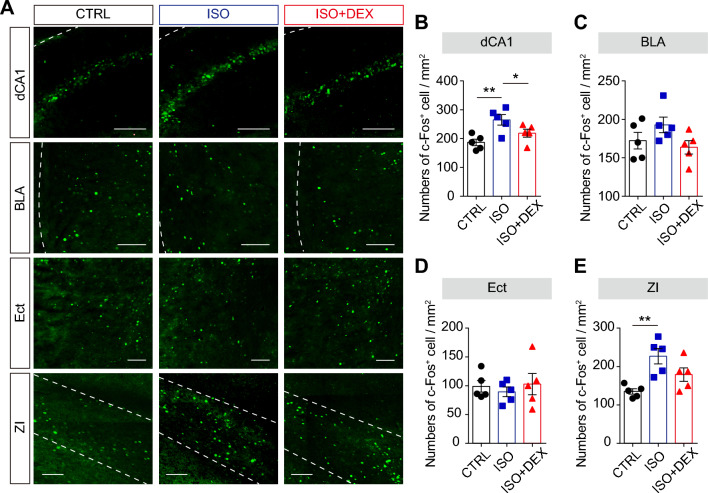
ISO-induced fear memory deficit is associated with the activation of ZI. (**A**) Representative images of c-fos immunostaining in the dCA1, BLA, Ect and ZI for control, ISO, ISO + DEX groups. Scale bar: 200 µm. (**B**) Number of c-fos-positive cells/mm^2^ in the dCA1, BLA, Ect and ZI for for control, ISO and ISO + DEX groups. n = 5 mice for each group. One-way ANOVA, dCA1: F(2, 12) = 7.082, **P < 0.01; ZI: F(2, 12) = 8.367, **P < 0.01; followed by post hoc Fisher’s LSD test, **P < 0.01, ****P < 0.0001. All data are expressed as mean ± SEM. Abbreviations: dCA1, dorsal CA1;BLA, basolateral amygdala; Ect, entorhinal cortex; ZI, zona incerta.

To identify specific brain regions that contribute to early life ISO-induced fear memory dysfunction, we quantified the expression of c-fos, a marker for neuronal activation, across several brain regions in response to fear memory retrieval. We selected the hippocampus, entorhinal cortex (Ect), basolateral amygdala (BLA), and ZI due to their links to memory and cognitive functions, particularly since the first three regions have been shown to be involved in inhalation anesthetic-induced neurotoxicity in animal studies^24–27^. The ZI was included because it has recently been identified as a crucial component of the fear memory circuitry^28–30^, despite limited research on its role in anesthetic-induced neurotoxicity. After exposing mice to the context of shock box, ISO mice showed significantly higher c-fos expression in the ZI compared to control mice (Fig. 2A,E). Notably, ISO + DEX mice exhibited a trend towards reduced c-fos expression compared to the ISO group, with the difference being marginally significant (Fig. 2A,E). Similar phenomena were observed in the dorsal hippocampus, but not in other brain regions such as Ect and BLA (Fig. 2A–D). Given that previous studies had demonstrated the involvement of the hippocampus in ISO-induced fear memory defects^24,25^, we focused our subsequent experiments on the ZI.

### ZI activation during fear memory retrieval in *ISO* and *ISO* + DEX mice

**Figure 3:**
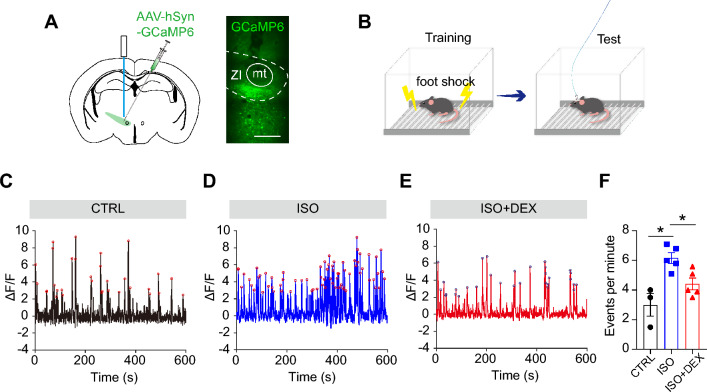
ZI activity during fear memory retrieval in ISO and ISO + DEX mice. (**A**) Schematic depicting the fiber photometry experiment used to record calcium activity from ZI neurons (left), and representative images displaying GCaMP expression (right). Scale bar: 200 µm. (**B**) Diagram illustrating the fiber photometry recording experiment. (**C**) Representative calcium dynamics of ZI neurons during retrieval of fear memory for the control group. Red circles indicate the positions of detected peak values for each Ca^2+^ transient event. (**D**) Representative calcium dynamics of ZI neurons during retrieval of fear memory for the ISO group. Red circles indicate the positions of detected peak values for each Ca^2+^ transient event. (**E**) Representative calcium dynamics of ZI neurons during retrieval of fear memory for the ISO + DEX group. blue circles indicate the positions of detected peak values for each Ca2 + transient event. (**F**) Comparison of frequency of Ca^2+^ transient events per minute among control (n = 3), ISO (n = 5) and ISO + DEX (n = 5) groups. Mann–Whitney test, *P < 0.05. All data are expressed as mean ± SEM.

We monitored the activity of ZI neurons by expressing genetically-encoded Ca^2+^ indicators (GCaMP6s) in the ZI (Fig. 3A). After three weeks of virus expression, these mice underwent fear conditioning training, and fiber photometry recordings were performed during the retrieval stage of fear memory (Fig. 3B). We found that ISO mice exhibited increased ZI neuron activity, as indicated by the frequency of Ca^2+^ events, compared to the control group. Furthermore, DEX treatment suppressed this heightened activity (Fig. 3C–F), which is consistent with the c-fos analysis results.

### Chemogenetic inhibition of ZI in *ISO* mice improved fear memory retrieval

**Figure 4:**
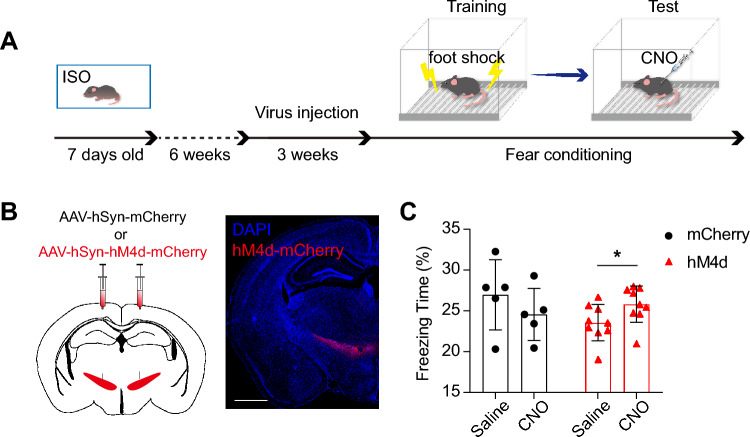
Chemogenetic activation and inhibition of ZI bidirectionally regulate fear memory. (**A**) Graphical representation of the sequence of behavioral paradigms. (**B**) Schematic of the Zi injection for the AAV virus and representative image showing the expression of hM4D. Scale bar: 1 mm. (**C**) Percentage of freezing time during the retrieval of contextual fear memory for mCherry (n = 5) and hM4d (n = 9) group. Paired t test, *P < 0.05. All data are expressed as mean ± SEM.

The aforementioned c-fos and Ca^2+^ activity results suggested that the inhibition of ZI was crucial for successful fear memory retrieval. To test whether inhibition of ZI could rescue ISO-induced fear memory deficit, we expressed chemogenetic inhibition tool hM4Di in the ZI (Fig. 4A,B), which are exclusively inhibited by designer drugs (DREADDs)^31^. In the ISO mice, Chemogenetic inhibition of ZI neurons through an IP injection of clozapine N-oxide (CNO, 4 mg/kg) significantly improved impaired retrieval of fear memory, as evidenced by increased percentage of freezing time (Fig. 4C). Thus, inhibition of ZI was sufficient to rescue ISO-induced fear memory deficits.

## Discussion

The potential long-term effects of anesthesia on cognitive development have been a subject of intense scrutiny, especially concerning neonates and infants^4,8^. Our study provides a comprehensive examination of the effects of isoflurane, a commonly used volatile inhalation anesthetic, on cognitive functions in mice. Furthermore, we delve into the potential protective role of dexmedetomidine against isoflurane-induced cognitive impairments.

Our study primarily focuses on the role of the zona incerta (ZI) in mediating cognitive and memory deficits due to early exposure to isoflurane. The ZI predominantly consists of GABAergic neurons and receives input from various brain regions, notably the central nucleus of the amygdala (CeA), which is intimately linked to emotional and memory processing^28^. Drawing from existing literature, the ZI has been identified as a crucial component of the fear memory circuitry^28–30^. The c-fos data suggests that early ISO treatment amplifies activity in the ZI and dCA1 regions during the fear conditioning test, rather than in the BLA or Ect. While the hippocampus has been previously confirmed to play a role in cognitive and memory deficits induced by early ISO exposure^24,25^, our study introduces the involvement of ZI activity for the first time. This proposition is further supported by calcium signal recordings, suggesting that the ZI could be a vulnerable region affected by ISO in the context of fear memory. Moreover, through optogenetic or chemogenetic techniques, some studies found that the activation of ZI neurons or their projections to PAG reduces the conditioned freezing response, while optogenetic suppression of these neurons enhances it^29,30^. These findings align with our chemogenetic manipulation results. Collectively, our results strongly suggest that the ZI may indeed be a key region mediating the cognitive impairments caused by ISO.

We also discovered that dexmedetomidine can alleviate the fear memory damage caused by early exposure to ISO, highlighting its potential protective role. This aligns with previous findings in rats^22,32,33^. Dexmedetomidine is a highly selective α2 adrenoceptor agonist known for its unique sedative properties that are associated with rapid arousal^34^. Past research has identified dexmedetomidine's antiapoptotic effects, which counteract neuronal death and cognitive impairments induced by isoflurane^22,32,33^. The antagonistic effects of the α2 adrenoceptor blocker, atipamezole, can negate the protective effects of dexmedetomidine^35^, suggesting that the α2 adrenoceptor might be a crucial target through which dexmedetomidine combats ISO-induced cognitive impairments. Notably, studies have shown that GABAergic neurons in the ZI express α2 adrenoceptors^36–38^, hinting that dexmedetomidine might directly act on the α2 adrenoceptors in the ZI region, exerting its effects during critical periods. In conclusion, the observed mitigation of isoflurane-induced fear memory deficits by dexmedetomidine offers an interesting perspective and suggests a potential avenue for therapeutic strategies.

While our study has shed light on the potential neural mechanisms underlying isoflurane-induced cognitive impairments and the protective role of dexmedetomidine, several questions remain. The precise molecular mechanisms through which isoflurane affects the ZI and how dexmedetomidine counteracts these effects warrant further investigation. Additionally, understanding the broader network dynamics involving the ZI and other brain regions during cognitive tasks could provide a more holistic view of the observed effects.

In conclusion, our study underscores the potential risks associated with early exposure to isoflurane and highlights the therapeutic potential of dexmedetomidine. The central role of the ZI in mediating these effects offers a promising target for future interventions. As the debate on the safety of anesthetics in pediatric populations continues, our findings provide valuable insights and directions for future research.

## Methods

### Animals

All experiments were conducted in accordance with relevant guidelines and regulations, and approved by the IACUC (Institutional Animal Care and Use Committee) of SIAT, Chinese Academy of Sciences. Wild type C57BL/6J Mice (Charles River, Beijing, China) Animals were maintained under a standard 12 h light/dark cycle (lights on at 8:00 A.M.) at a constant temperature of 23 ± 1 °C, with food and water available ad libitum. All methods used in this study conformed to the ARRIVE guidelines.

### Nental isoflurane exposure paradigm

Mice pups at 7 days old were randomly assigned to three groups: control, isoflurane (ISO), and isoflurane + dexmedetomidine (ISO + DEX). In the ISO group, pups were anesthetized with isoflurane (0.75%) for 6 h. Mice were exposed to 0.75% isoflurane (RWD Life Science Co., R510-22–10) and anesthetized deeply for 6 h. Physiologic variables were assessed, and body temperature was controlled at 36.5 °C. For the ISO + DEX group, pups received a DEX treatment (intraperitoneally at 75 µg/kg) 20 min prior to isoflurane anesthesia. Dexmedetomidine (75 µg/kg, i.p. Cat#: 138445-3, Orion Pharma, Finland) was administered before isoflurane anesthesia. Control mice were placed in the anesthesia box for 6 h without exposure to isoflurane or dexmedetomidine, but under conditions identical to those of the isoflurane group. After the treatments, mice were housed in an SPF environment for 7 weeks. Then they were subjected to fear conditioning training. Memory retrieval tests were conducted on the following day.

### Fear conditioning

Seven weeks after anesthesia, the mice underwent fear conditioning in chambers made of transparent acrylic, each measuring 30 cm in height, 25 cm in width, and 25 cm in depth. The grid floors, composed of 4 mm diameter stainless-steel bars spaced 16 mm apart, were connected to a shock delivery system. The chambers were wiped with 75% ethanol before and after each session.

After a 3-min baseline exploratory period in the chambers, all mice underwent five training trials. In each trial, five pure tones (4 kHz, 65 dB, 30 s each) were presented, co-terminating with a 2-s, 1-mA foot shock, delivered at 1-min inter-trial intervals (ITI). Freezing, defined as the absence of any movement except for respiration, was recorded and scored for each animal. The percentage of freezing was calculated using the formula 100 × f/n, where f is the time of freezing per mouse and n is the total observation time per mouse. The time window used for quantifying the acquired fear response was from the first tone presentation to the end of the last shock.

Post-training, mice were tested for their retrieval of the fear response by placing them back in the shock chambers. During this test, no shocks were delivered, but the same pure tones (4 kHz, 65 dB) were presented. Each tone lasted for 30 s with an ITI of 30 s. Freezing behavior was scored during a 3-min exploratory period. For fiber photometry recording, the test lasted for 10 min.

### Virus preparation

The viruses were produced by Taitool Bioscience (Shanghai, China) and included the following: AAV2/9-hsyn-Gcamp6s (S0225), AAV2/9-hsyn-mCherry (S0238-9), and AAV2/9-hsyn-hM4d-mCherry (S0279). Prior to dilution, the viral vector titers ranged from 0.8 × 10^13^ to 2 × 10^13^ viral particles/ml. Following preparation, the final titers of these vectors were adjusted to fall within the range of 2 × 10^12^ to 6 × 10^12^ viral particles/ml.

### Stereotaxic surgeries

Mice were anesthetized with intraperitoneal (i.p.) injections of pentobarbital (80 mg/kg) and positioned in a stereotaxic frame (RWD Life Science Co., LTD., Shenzhen, China, Cat No. 68019). The virus was injected into the ZI (AP: − 1.95, ML: + 1.0, DV: − 4.45) using a syringe pump (KD Scientific, Massachusetts, US) at a rate of 50–70 nL/min for a total volume of 200–400 nL. For calcium imaging experiments, the optic fibers were first mounted on a specialized holder and secured to the stereotaxic arm to ensure vertical alignment. Using bregma as the reference point, the fibers were carefully positioned at the ZI coordinates used for the virus injection, with the fiber tip placed 100 μm above the injection site. These fibers were securely anchored to the skull using light-cured dental resin. Animals were allowed to recover for at least three weeks before behavioral experiments.

### Fiber photometry recording

Three weeks post-surgery, mice in both the ISO and ISO + DEX groups first underwent fear conditioning training, fiber photometry recording was performed during the retrieval stage for at least 10 min using a commercial fiber photometry system (ThinkerTech Inc., Nanjing, China). Signals were collected at a sampling frequency of 100 Hz and analyzed using custom-written MATLAB code. A 470 nm and a 405 nm LED light (each 30–40 μW) were channeled through a single implanted optic fiber, and fluorescence signals from both wavelengths were recorded simultaneously. The 405 nm signal, which is not calcium-dependent, was used to correct for autofluorescence, bleaching, and fiber bending artifacts by subtracting the 405 nm signal from the 470 nm signal.

The median of the entire trace was defined as the baseline fluorescence value (F0). The normalized change in fluorescence (ΔF/F) was calculated using the formula ΔF/F = (F – F_0_)/F_0_. Ca^2+^ transient events were identified as peaks in ΔF/F that exceeded 1.96 standard deviations of the entire trace.

### Histology

Mice were euthanized with an overdose of pentobarbital sodium and transcardially perfused with phosphate-buffered saline (PBS, pH 7.4) followed by 4% paraformaldehyde (PFA). Brains were dissected and fixed in 4% PFA for 1–2 h at room temperature. After dehydration in 30% sucrose for 48–72 h, brain tissues were embedded in Tissue-Tek OCT compound (Sakura) and frozen at − 20 °C before sectioning. Brains were cut into 40-μm sections using a cryostat (Leica).

For the detection of c-fos expression, sections matching the regions of the zona incerta, dorsal hippocampus, entorhinal cortex, and basolateral amygdala were selected based on the stereotaxic atlas of the mouse brain. Initially, the sections were washed with PBS. They were then incubated for 2 h in a blocking buffer composed of 10% goat serum in 0.7% Triton X-100 (PBST). Primary antibodies, specifically a rabbit monoclonal anti-c-fos (dilution 1:200, Cat# 2250, Cell Signaling), were prepared in a staining buffer containing 0.35% Triton X-100 and 5% goat serum. The sections were incubated with this antibody solution overnight at 4 °C. The next day, the sections were washed three times with PBS, each wash lasting 10 min. They were then exposed to secondary antibodies, Alexa Fluor 488 goat anti-rabbit IgG (dilution 1:500, ab150077, Abcam), prepared in the staining buffer, for 2 h at room temperature. Subsequently, sections were washed with PBS and co-incubated with DAPI at room temperature for 10 min. Following a final wash with PBS, the sections were mounted onto slides.

Images of the stained sections were captured using a virtual slide microscope (Olympus, VS120-S6-W). Initially, sections were scanned under brightfield at 2× magnification for an overall assessment. The focal points were then selected for multiple-point focusing on the target brain areas. Micro-adjustments were made based on the DAPI signal to achieve optimal imaging. Consistent exposure and excitation parameters were maintained across different mice. Finally, images were captured at 10× magnification.

The images were exported to ImageJ for further analysis. After setting the scale according to the actual dimensions, regions of interest (ROIs) in the target areas (ZI, dCA1, BLA, Ect) were selected according to the stereotaxic atlas of the mouse brain. Cell counting was performed under the same analysis parameters across different mice.

### Enzyme-linked immunosorbent assay (ELISA)

To determine corticosterone levels, blood samples were drawn from the tail vein and immediately placed in ice-cold tubes containing heparin, a protease inhibitor cocktail, and ethylenediaminetetraacetic acid (EDTA). Following centrifugation at 2000 rpm for 10 min at 4 °C, the resulting supernatants (serum) were collected, aliquoted, and stored at − 80 °C until further analysis. Corticosterone levels were quantified using an ELISA kit (Abcam, ab108821) following the manufacturer's standard protocol.

### Statistical methods

Normality of the data was assessed using the Shapiro–Wilk test. For group comparisons, Student’s t-test and ANOVA were used for normally distributed data. For non-normally distributed data, non-parametric tests such as the Mann–Whitney test were employed. All statistical data can be found in the figure legends. Statistical significance was set at *p* < 0.05. Data are presented as means ± SEM.

## Data Availability

All raw data, code, and materials used in this study are available from the corresponding author upon reasonable request.
